# The Impact of Nucleotide Sequence Analysis on Meningococcal Vaccine Development and Assessment

**DOI:** 10.3389/fimmu.2018.03151

**Published:** 2019-01-15

**Authors:** Martin Christopher James Maiden

**Affiliations:** Department of Zoology, University of Oxford, Oxford, United Kingdom

**Keywords:** *Neisseria meningitidis*, conjugate polysaccharide vaccines, outer membrane vesicle vaccines, population biology, herd immunity, efficacy

## Abstract

Since it became available as a routine tool in biology, the determination and analysis of nucleotide sequences has been applied to the design of vaccines and the investigation of their effectiveness. As vaccination is primarily concerned with the interaction of biological molecules with the immune system, the utility of sequence data is not immediately obvious and, indeed, nucleotide sequence data are most effective when used to complement more conventional immunological approaches. Here, the impact of sequencing on the field of vaccinology will be illustrated with reference to the development and implementation of vaccines against *Neisseria meningitidis* (the meningococcus) over the 30-year period from the late-1980s to the late-2010s. Nucleotide sequence-based studies have been important in the fight against this aggressive pathogen largely because of its high genetic and antigenic diversity, properties that were only fully appreciated because of sequence-based studies. Five aspects will be considered, the use of sequence data to: (i) discover vaccine antigens; (ii) assess the diversity and distribution of vaccine antigens; (iii) determine the evolutionary and population biology of the organism and their implications for immunization; and (iv) develop molecular approaches to investigate pre- and post-vaccine pathogen populations to assess vaccine impact. One of the great advantages of nucleotide sequence data has been its scalability, which has meant that increasingly large data sets have been available, which has proved invaluable in the investigation of an organism as diverse and enigmatic as the meningococcus.

## Introduction

The 40 years following the introduction of the Sanger dideoxy method in 1977 (1) saw a revolution in biology, which was driven by the improvement of nucleotide sequencing technologies. At the start of this period, determining a DNA or RNA sequence was a highly specialized task, which was undertaken in a very few laboratories most often using their own home-made equipment and reagents. Only individual genes or viruses could be sequenced, and then at great expense. By 2018, nucleotide sequencing was conducted on an industrial scale, employing mass-produced reagents and highly automated equipment, often in large factory-like installations. Complete genome sequences were assembled routinely for tens of thousands, even hundreds of thousands of organisms, including those with the largest genomes. Major advances had also been made in the computer equipment and software available to interpret the data produced, although it is fair to say that while issues of data generation were for all practical purposes resolved, data interpretation remained a major hurdle. In common with most areas of biology, vaccine development, and evaluation were transformed by these advances and this transformation will be illustrated here using meningococcal vaccines as an example.

Many of the most successful bacterial and viral vaccines were developed in the mid- to late- twentieth century, without recourse to the detailed genetic information that nucleotide sequencing provides; however, most of these conventionally-developed vaccines targeted antigenically stable pathogens, such as the smallpox virus, or those that rely on a single, stable, molecule for their pathogenicity, such as *Corynebacterium diphtheriae* (diphtheria) and *Clostridium tetani* (tetanus). The bacterium *Neisseria meningitidis*, the meningococcus, very nearly falls in to this category, as almost all invasive meningococcal disease (IMD) is caused by bacteria that express one of only six capsular polysaccharide antigens, referred to as serogroups A, B, C, W, X, and Y. Polysaccharide and, especially, protein-conjugate polysaccharide, vaccines are effective in protecting against disease for five of these (serogroups A, C, X, W, X, and Y) and provide a means of eliminating most IMD worldwide (2). Unfortunately, however, vaccines directed against meningococcal serogroup B polysaccharide have not been developed. This is a consequence of poor immunogenicity and fear of inducing host autoimmune disease, due to similarities of the serogroup B polysaccharide with human antigens (3, 4). The search for alternative antigens that target serogroup B meningococci has been complicated by the high variability of virtually all other possible meningococcal vaccine components. Ever-increasing volumes of nucleotide sequence data have been used in this search.

As successful immunization is almost always a population process, population studies based on nucleotide sequences have important applications in the development, deployment, and evaluation of vaccines. Here I shall describe how nucleotide sequence technologies have contributed to vaccine development and the assessment of vaccines, outlining the development, and implementation of meningococcal vaccines from the late 1970s to the time of writing.

## Structure and Variation in Individual Antigens

Determining the nucleotide sequence of a gene encoding a vaccine antigen permits the deduction of a wealth of information concerning the protein and enables a wide range of follow-up studies. For example, the PorA protein was established as a potential vaccine component for “serogroup B substitute” vaccines (i.e., vaccines developed as an alternative to those including serogroup B capsular polysaccharide as an antigen) in the late 1970s and early 1980s, on the basis of immunological and biochemical studies (5, 6). The cloning and sequencing of the *porA* gene in 1991 provided much additional information, for example confirming that it was a typical porin related to those found in the gonococcus (7). The meningococcal and gonococcal sequences were used to design oligonucleotide primers for the then new PCR technique, enabling the amplification, and sequencing of the *porA* gene from multiple meningococcal isolates (8), illustrating how sequence technologies lead to cumulative gains in knowledge. These comparative studies enabled structural models to be proposed and established that the antigenic variability identified by subtype-specific monoclonal antibodies was mostly determined by the peptide sequences of two major variable regions (VR1 and VR2) and one less variable region (VR3 or sVR) of the PorA protein. Combining sequencing and immunological studies enabled these antigens and their interaction with immune molecules to be defined (8–10).

The combination of PCR amplification and Sanger sequencing enables multiple variants of a given gene to be characterized accurately and rapidly at high volume. This permits the antigenic variability of a given protein vaccine component to be established. In the case of meningococcal PorA, the variants of which were initially identified with monoclonal antibodies (11), an ever-increasing diversity of variants have been identified by sequencing in the past 30 years. This required that the original nomenclature, which was based on monoclonal antibody reactivity, needed to be replaced with an updated nomenclature scheme that was based on the peptide sequences themselves, rather than the antibodies that reacted with them (12). It was important that this scheme was backwards compatible with the designations of the established antibody-based system. The scheme needed to be infinitely expandable, and to this end variant numbers were used, which could be added to as each new variant was discovered. At the time of writing at total of 324 peptide variants had be defined or PorA VR1 and 906 for PorA VR2. An advantage of sequence-based nomenclature is that the sequences can be recorded on open-access web based databases (e.g., https://pubmlst.org/neisseria/PorA/), enabling easy access to the nomenclature (13). Similar approaches have since been used to catalog the variation of a number of meningococcal vaccine candidates including: factor H binding protein (fHbp) (14); the ferric enterochelin receptor, FetA (15); *Neisseria* adhesin A (NadA) (16); and the heamoglobin receptor (HmbR) (17).

**Figure 1:**
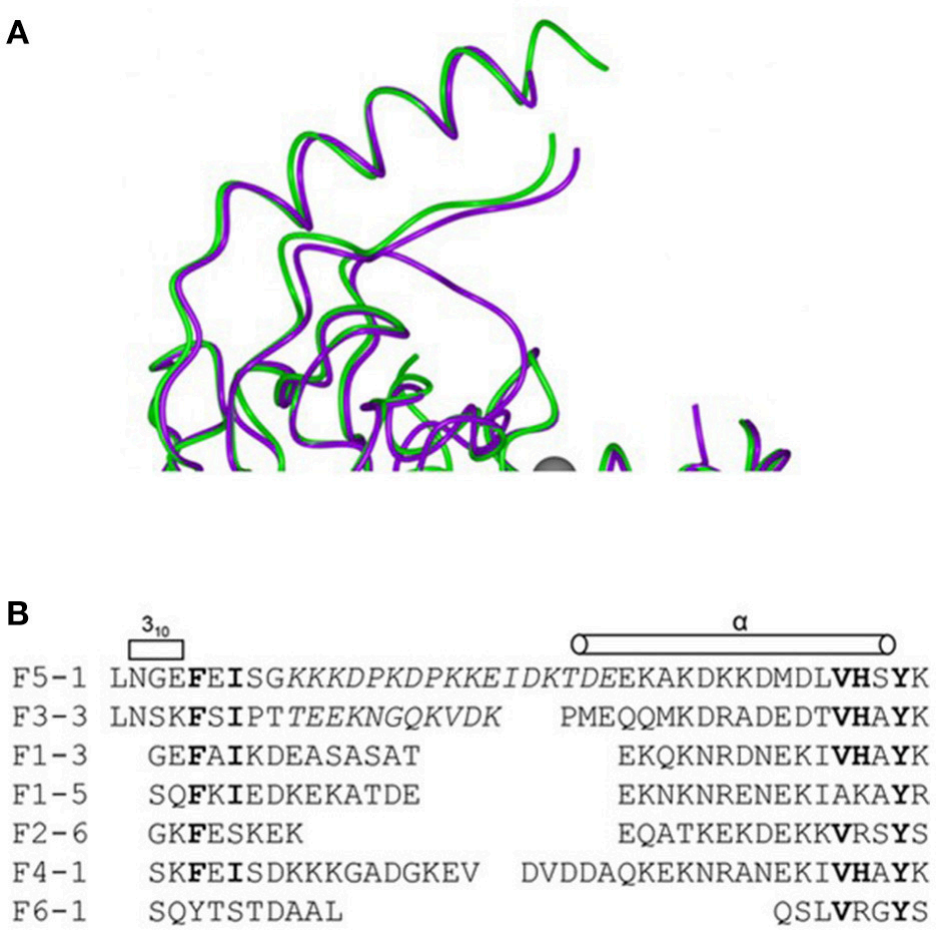
Combining sequence data with and structural information to define variation in a meningococcal antigen. The structure and alignment of the FrpB (FetA) hypervariable sequence regions. **(A)** Superposition of two hypervariable regions F5-1 purple, F3-3 (A chain) green. **(B)** Sequence alignment of the HR regions from the major FrpB variants. Reproduced without modification from Saleem et al. (20) under CC BY.

In addition to describing the nature and extent of variation of genes, including those encoding vaccine antigens, sequence analyses help to reveal the mechanisms whereby this variation occurs. In the case of PorA, the immunogenic VRs are relatively short continuous sequences, encoding surface-exposed parts of the porin structure (18), which vary by point mutation, insertion, and deletion. Each of these processes can have an impact on the binding of immune molecules such as antibodies to the expressed protein. These impacts have been assessed by a combination of sequence comparison, biochemical, structural, and immunological analyses (19, 20) (Figure 1). Sequencing studies have also established that protein antigen expression can be influenced by the sequence of control regions, with polynucleotide tracts playing an important role in the expression of a number of meningococcal antigens (21). Another mechanism of variation that sequencing studies identified is the exchange of genetic material *via* horizontal genetic transfer (HGT), and the PorA protein was one of the first bacterial genes in which this was extensively documented (22). The recognition of the importance of HGT in bacterial evolution came from studies of antibiotic resistance and vaccine antigens in the gonococcus (23), meningococcus (22), and pneumococcus (24), with a major impact on our understanding on bacterial population biology and evolution (25).

## Bacterial Population Biology and Evolutionary Studies

Knowledge of the extent of variation in vaccine antigens inevitably poses questions of how this variation comes about and how it moves through the population. Both topics have important implications for vaccine design, as it is essential to know how effective a given vaccine will be once it has been introduced and how easily vaccine escape variants might arise and spread. Population studies of the pathogenic *Neisseria*, both the meningococcus and *Neisseria gonorrheae* (the gonococcus), have played a major role in developing bacterial population genetics. In the case of the meningococcus, these studies have also been central in the design and implementation of new vaccines.

Bacterial population studies predate the sequencing era, with seminal investigations of the meningococcus using a combination of multi-locus enzyme electrophoresis (MLEE) (26) and immunological typing (27–29), establishing fundamental concepts that have been built on subsequently by sequence-based investigations. MLEE investigations conducted on isolates from cases of invasive disease, showed that a limited number of groups of closely related organisms, known as “clones” or “genetic lineages,” each of which was associated with particular antigenic characteristics, were predominant causes of disease (27, 29–32). By contrast, meningococci isolated from asymptomatic carriers were much more diverse (33, 34). This led to the concept of “hypervirulent” or “hyperinvasive” meningococci: persistent genotypes that were especially likely to cause IMD (35). One such lineage, associated with electrophoretic type 5 (ET-5), caused an international outbreak of serogroup B IMD (30), resulting in the development and implementation of specific outer membrane vesicle (OMV) vaccines generated against the local outbreak strain in Norway (36) and Cuba (37). This approach was repeated in New Zealand 15 years later (38).

**Figure 2:**
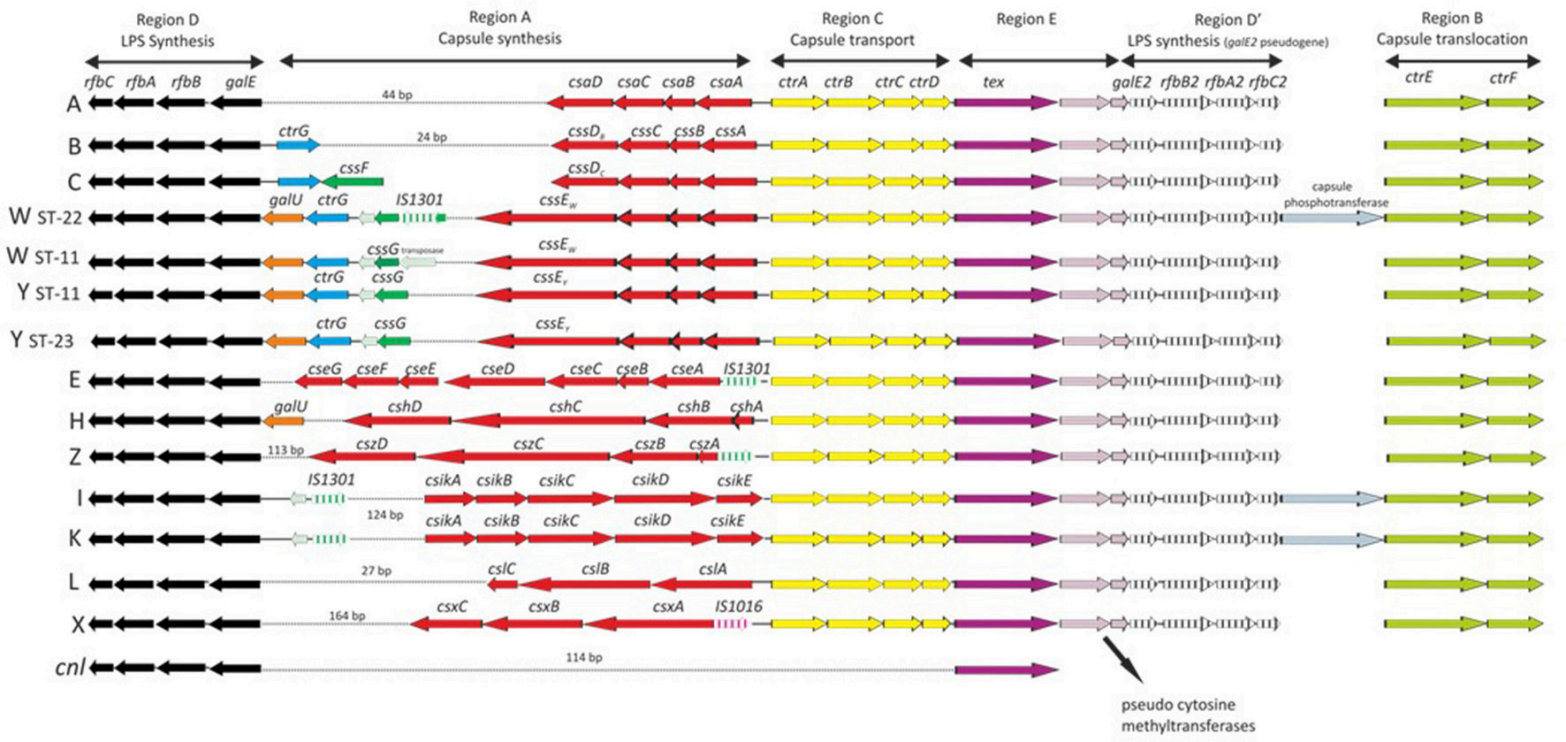
Genetic organization of the cps locus among *Neisseria meningitidis* serogroups revealed by nucleotide sequencing. Serogroups A (*N. meningitidis* isolate Z2491); B (H44/76); C (FAM18, 053442, and 29013); W (α275); W (WUE171); Y (α162); Y (WUE172); E (α707); H (29031); I (29043); K (29046); L (WUE3608); X (α388); and Z (WUE173); and a capsule null (*cnl*) isolate. Letters on left represent serogroups. Arrows depict gene orientation. Reproduced without modification from Harrison et al. (43) under CC BY.

The high levels of HGT observed in genes encoding antigens (22, 39) and antibiotic resistance (23), sometimes involving inter-species transfer (23, 40), are also a observed in “housekeeping” genes: those expected to be subject to stabilizing selection for the conservation of metabolic function (41). This demonstrates the important of HGT in bacterial evolution generally, and especially in the *Neisseria*. One consequence of this is the a range of population structures observed in the meningococcus (25). MLEE technology was difficult to implement and compare across studies and, as sequencing technology developed, it was replaced by a nucleotide sequence-based approach, multilocus sequence typing (MLST), which indexed the variation in seven ~400 bp fragments of housekeeping genes: *abcZ*; *adk*; *aroE*; *fumC*; *gdh*; *pdhC*; and *pgm*. MLST was much easier to implement at scale and had the advantage of being easily portable among laboratories (42). Originally developed for *Neisseria meningitis*, MLST has had wide application to many bacterial species and, in combination with web-accessible databases, has become a widely used method, with applications in, for example: evolutionary studies; epidemiology; population biology; and taxonomy (42). MLST studies enabled genetic lineages to be defined in terms of groups of related STs called clonal complexes (ccs), which were named after the ST that was used to define that complex. Thus, ST-11 is the defining sequence type for clonal complex 11 (cc11), also known as the “ST-11 complex.” These molecular designations have been incorporated with other typing schemes such as serogrouping (for capsule) (43) (Figure 2), subtyping (for PorA) (12) and typing of the FetA antigen (Figure 1) (15) into a standardized typing nomenclature with the form: B: P1.19,15: F5-1: ST-33 (cc32), referring to: group (in this case B); PorA VR1 and VR2 type (P1.19,15); FetA VR type (F5-1); sequence type (ST-33); and clonal complex (cc32), respectively (44).

## The Population Approach to Vaccine Assessment

High-throughput sequencing approaches, which permit the characterization of hundreds or thousands of bacteria isolates at multiple loci, enable a population genetic approach to be taken to the assessment of vaccination programs. In the case of *N. meningitidis*, these approaches played a central role in understanding the impact of conjugate polysaccharide vaccines, which targeted the meningococcal capsular antigen (2). In late 1999, just after the publication of the MLST approach in 1998, the United Kingdom (UK) Department of Health implemented a novel monovalent serogroup C conjugate polysaccharide vaccine, in response to a dramatic increase in serogroup C meningococcal disease in the UK (45). The protein-polysaccharide vaccines had significant advantages over the older “plain” polysaccharide vaccines, in that the conjugate vaccines generated more effective immune responses. This included eliciting protective responses in younger individuals, affinity maturation, and a memory response, none of which were generated by the “plain” polysaccharide vaccines (46). A combination of the urgency of the public health response and the epidemiology of IMD meant that the new meningococcal C conjugate (MCC) vaccines were implemented without the benefit of a phase III efficacy trial (2). This led to several uncertainties, not least that it was unknown whether the vaccine would affect asymptomatic carriage. Although the prospect of directly preventing disease was wholly positive, there was a concern that population effects might lead to the emergence of vaccine escape variants—variants of the epidemic clone that had acquired a different capsule by HGT (47). Investigation of this required monitoring, not only disease isolates to establish impact on disease, but also carried meningococci, during vaccine implementation period.

The United Kingdom Meningococcal Carriage (UKMenCar) study was established to measure the impact of MCC on carriage and surveyed 47,765 teenagers from 1999 to 2001, immediately before and for 2 years after vaccine implementation (48–50). Oropharyngeal swab samples and conventional culturing were used to isolate meningococci from the throats of the subjects. In combination with phenotyping, high-throughput single-locus sequencing of the capsular region combined with MLST analysis enabled the nature and status of the serogroups to be determined, together with the clonal complex of each isolate. These data showed that, before vaccine implementation, the point prevalence of carriage of the epidemic-causing meningococcus (C:cc11) was low (0.31% of individuals), which is characteristic of a highly hyperinvasive meningococcus. In 2001, 2 years after implementation, this carriage rate decreased by 80% (to 0.04% of individuals). As a proportion of the meningococci isolated, the C:cc11 epidemic strain dropped by nearly 90%, from 1.83 to 0.21%, with a coincident reduction in the proportion expressing the capsular antigen of around 50% (48). This resulted in a significant herd immunity (protection) of the unvaccinated (51), which was fortunate as the initial vaccination schedule used for infants (2, 3, and 4 months) provided no direct protection more than a year after immunization (52). The vaccination program had no significant effect on the carriage of other genotypes and serogroups, although there was some evidence for secular changes over the period of the surveys (50).

The success of the monovalent MCC vaccines, demonstrated to be in large degree due to their ability to induce herd immunity, catalyzed interest in developing a similar vaccine to target the periodic very large epidemics seen in the African Meningitis Belt. These represent one of the most important manifestations of IMD globally and were first described by Lapeyssonnie (53). Since that time, repeated serogroup A epidemics had been observed, with especially large outbreaks in the late 1990s (54). The meningitis vaccine project (MVP) was established in 2001 as a partnership between PATH (formerly the Program for Appropriate Technology in Health) and the World Health Organization (WHO), funded by the Bill and Melinda Gates foundation. The aim of the MVP was the elimination of meningococcal epidemics in Africa by means of an affordable serogroup A protein-polysaccharide conjugate vaccine. Employing an innovative public-private approach with northern and southern partners, this goal was achieved, with the vaccine PsA-TT, “MenAfriVac^®^,” introduced in December 2010, with a plan to immunize all African Meningitis Belt countries by 2013 (55, 56).

The Meningococcal Carriage in Africa (MenAfriCar) consortium was established in 2008 to measure the impact of the PsA-TT vaccine on carriage and the impact of herd immunity (57). Up until that time, knowledge of the carriage of meningococci in this region was incomplete, with a number of studies undertaken at various times employing a variety of techniques. This limited the collation of consistent information and a wide range of carriage prevalence rates (3–30%) had been reported (58). The MenAfriCar consortium aimed to conduct large-scale carriage surveys across the meningitis belt before and after the introduction of the PsA-TT vaccine using consistent methods. Risk factor data for carriage were simultaneously collected, as in the UKMenCar surveys, although the risk factors included were somewhat different from those seen in the UK (59, 60). One unknown was the impact of the carriage of other *Neisseria* species, especially *Neisseria lactamica*, which is commonly isolated from individuals in Africa (61). This was challenging as members of the genus are very similar: they are poorly distinguishable by 16SrRNA sequencing (62), for example. The large number of isolates that would have to be processed by laboratories in resource-limiting environments presented further challenges that were met by the exploitation of nucleotide sequence-based approaches, made possible by the availability of genomic technologies.

The MenAfriCar surveys employed a combination of conventional culture, biochemical, and sequence-based methodologies. As in UKMenCar surveys, oropharyngeal swab samples were collected and cultured on selective media with putative *Neisseria* identified using biochemical tests in local laboratories (60). From these cultures, boiled cell preparations were made, which were shipped to Oxford where the molecular analyses were performed (61). To solve the speciation problem a novel assay was designed. This took advantage of an extended MLST scheme, ribosomal MLST (rMLST), which indexes the 53 genes encoding ribosomal proteins, and enables bacterial isolate characterization “from domain to strain” (63). From the rMLST sequences extracted from WGS data from 44 isolates of diverse species, a 413 bp fragment of the *rplF* gene was identified, the sequence of which was diagnostic for each *Neisseria* species. This fragment could be amplified and sequenced at high-throughput, enabling *Neisseria* species identification which was rapid, accurate, and cost-effective (64). The capsule genes were identified with a real time PCR assay and fine typing performed by sequencing the *porA* and *fetA* loci (60).

The MenAfriCar studies showed great diversity of meningococcal carriage across the belt and over time, which differed from the patterns of carriage typically observed in high-income countries with temperate climates, where carriage is more consistent (65). The age profiles of carriers was also different, with meningococcal carriage highest in individuals aged 5–14 (60), rather than in adults and adolescents as typically seen elsewhere (65). Transmission among children within households was shown to be important (66), again different from other settings, where social interactions outside the family are important (59). There was also much more diversity in the non-meningococcal *Neisseria* species isolated, the carriage of which was also age-related (61). This different dynamic was consistent with the unique epidemiology observed in African meningitis belt countries, and with the idea that the occurrence of seasonal epidemics in the meningitis belt were dependent on the transmission of epidemic clones.

**Figure 3:**
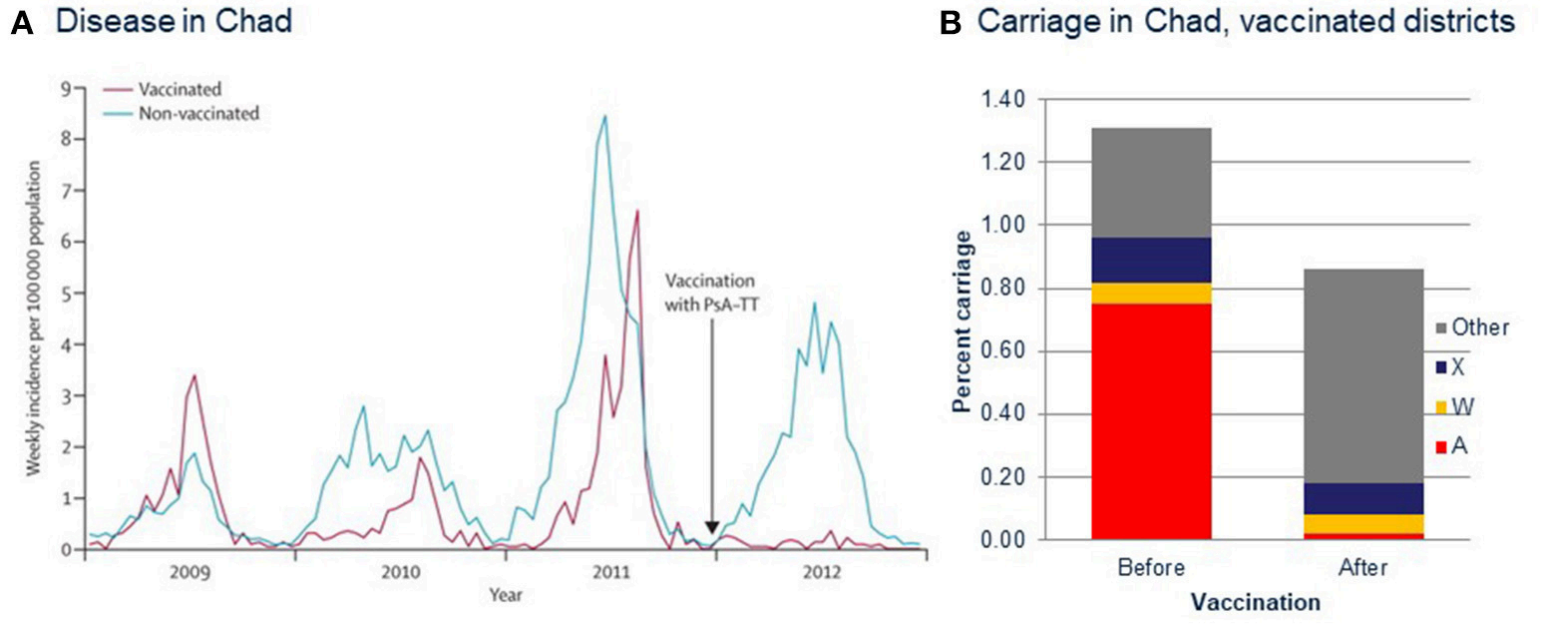
Measuring vaccine efficacy in the meningitis belt during PsA-TT (MenAfriVac) introduction in Chad using genomic sequencing techniques. **(A)** Incidence of reported cases of meningitis in Chad, 2009–2012. Vaccination with PsA–TT was undertaken in patients aged 1–29 years at the end of 2011 (arrow). PsA–TT=serogroup A meningococcal polysaccharide–tetanus toxoid conjugate vaccine. **(B)** Carriage rates of meningococci of different serogroups before and after vaccination. **(A)** Reproduced from Daugla et al. (68) without modification under CC BY.

Most of the counties in which PsA-TT was introduced including the first, Burkina Fasso, were not experiencing an epidemic of serogroup A IMD at the time of introduction, making assessment of the herd effects of the vaccine difficult, although effects on disease and carriage rates were consistent with such effects (67). An epidemic in Chad during the introduction there, however, enabled a direct demonstration of efficacy against IMD and carriage of the epidemic strain (68) (Figure 3). The rollout of the vaccines in different districts in over two epidemic years, combined with the MenAfriCar sampling and sequence-based isolate characterization protocols, demonstrated high efficacy of the vaccination against both IMD and carriage (68). Samples collected in this study also demonstrated that even at genomic levels of isolate characterization, there were no consistent differences between carried and invasive meningococci (69).

The marked successes of the conjugate vaccines in different settings provided the prospect of a “meningitis free world,” so long as an effective group B vaccine could be developed (70); however, no vaccine against the meningococcal serogroup B polysaccharide, either plain or protein-conjugate, has been developed to the time of writing and none was under development (3). This was due to a combination of the poor immunogenicity of the antigen combined with fears of the induction of auto-immunity, as a consequence of its similarity of the antigen to human neural polysaccharides (4). Consequently, even with the prospect of conjugate polysaccharide vaccines that target serogroups A, C, W, X, and Y (71), there is a need for alternative or “substitute serogroup B” vaccines, if IMD is to be comprehensively prevented (3).

## Genomic Disease Surveillance: Understanding and Combatting Virulence and Vaccine Escape

**Figure 4:**
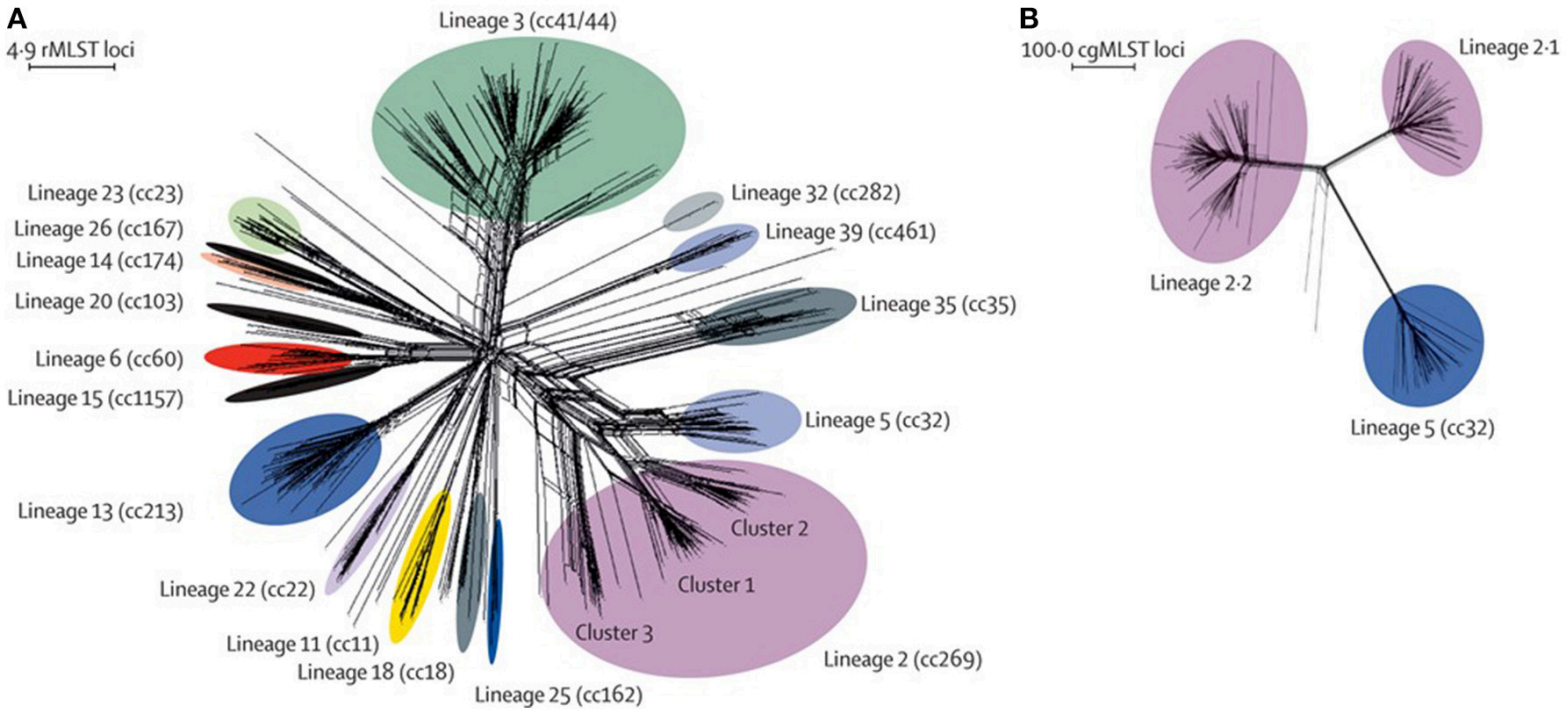
Genetic diversity of disease-associated meningococcal isolates in the Meningitis Research Foundation Meningococcus Genome Library for England and Wales **(A)** Neighbor-Net graph showing the relationships of all 498 rMLST profiles (ribosomal sequence types) within the 899 isolates available for epidemiological years 2010–2011 and 2011–2012. **(B)** Relationships among isolates belonging to lineage 2 (cc269; *n* = 171) and lineage 5 (cc32) isolates (*n* = 42) assessed with 1605 cgMLST loci. This analysis illustrates the improved resolution achieved with cgMLST for the substructures within and between lineages, compared with rMLST. rMLST=ribosomal multilocus sequence typing. cc=clonal complex. cgMLST=core-genome multilocus sequence typing. Reproduced without modification from Hill et al. (73) Under CC BY.

The development and validation of next generation sequencing approaches for the determination of high-quality draft WGSs of meningococci (72), led to the establishment of the Meningitis Research Foundation Meningococcus Genome Library (MRF-MGL) (73) (Figure 4). This repository contained the WGS data for all meningococci isolated from cases of IMD in the UK. The MRF-MGL provides the opportunity to identify and react to IMD outbreaks in real time or near real time. An example of this is the reaction of the UK public health authorities to a serogroup W IMD outbreak. From the early 2010s onwards, coinciding with the establishment of the MRF-MGL, there was a year-on-year increase in cases of serogroup W meningococcal disease, which data from the MRF-MGL showed to be W:cc11 (74). A similar increase had been observed a decade before (75), just after the successful introduction of the MCC vaccines, causing some alarm (76). The former increase had been associated with the global spread of a particular W:cc11 strain after the Hajj pilgrimage and had fortunately been transitory (77); however, the epidemiology of the cases in the 2010s was somewhat different, leading to the concern that this might be a different circumstance and that a larger epidemic might occur (74). A WGS study of a global collection of 750 diverse cc11 meningococci demonstrated that, although the cases in the UK were indeed related to the Hajj-associated isolates, they were much more closely related at the WGS level to W:cc11 meningococci that had caused large-scale epidemic outbreaks in South America (78). In combination with other epidemiological information, these data formed the basis of a decision to implement tetravalent A, C, W, Y conjugate vaccines into the UK immunization program for teenagers (79). Certainly, this was the first use of genomics to change national vaccination policy for meningococci and perhaps for any organism.

## Genome Sequencing Vaccine Design and Assessment

The availability of whole genome sequences (WGS) of bacterial pathogens also provided novel opportunities in the development of vaccines. In the case of the meningococcus, the first meningococcal WGSs, from bacterial isolates MC58 (80) and Z2491 (81) both published 2000 played a role in the development of two “serogroup B substitute” vaccines: Bexsero® (4CMenB, developed in Siena, Italy) (82); and Trumenba^®^ (LP2086, developed in Pearl River, USA) (83). In the case of the Bexsero® vaccine a “reverse vaccinology” (84) approach was adopted using the MC58 genome sequences as its starting point. In contrast to more conventional approaches, which took the interaction of a bacterial isolate as a starting point, reverse vaccinology started by predicting potential vaccine antigens (i.e., surface-exposed proteins) from a genome sequence, and then assessing these in animals. This identified three potential targets, fHbp, NadA, and NHBA that were eventually included in the final Bexsero® formulation, along with the MenNZB OMV vaccine, made against the New Zealand outbreak strain (38). Interestingly, a more conventional vaccine development approach, but which did use sequence information from the Z2498 meningococcal isolate to find the gene sequence from protein sequences, identified the fHbp protein (also known as LP2086) as an important vaccine candidate (83).

**Figure 5:**
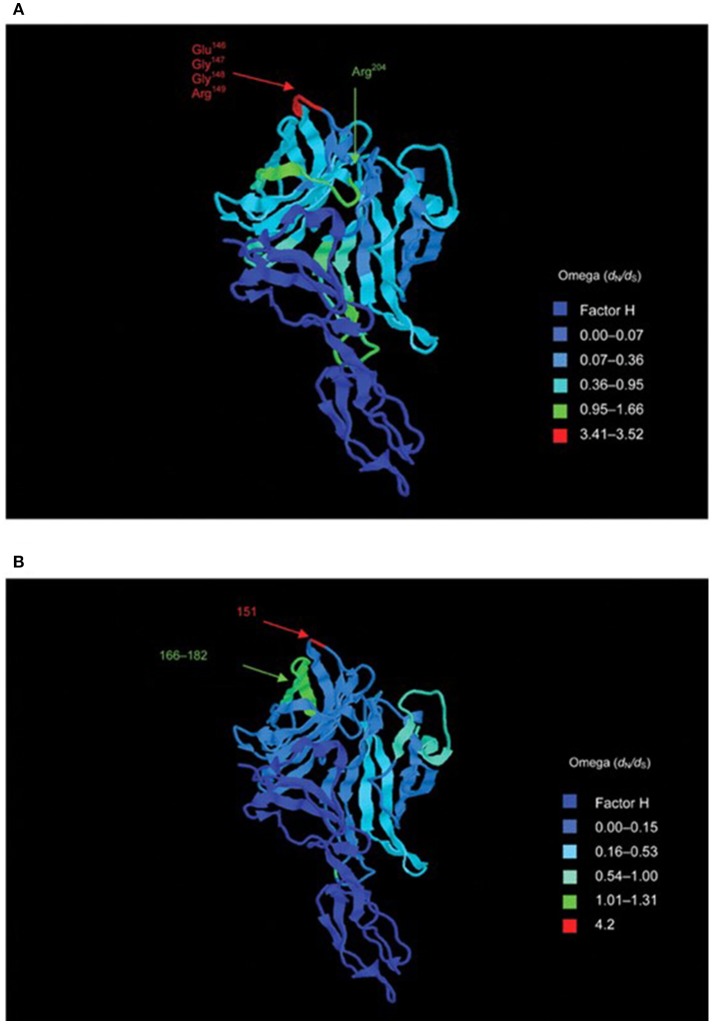
Structure of the fH–fHbp complex, with temperature coloring using per-site point estimates of selection (ω) for **(A)** subfamily B/variant 1 sequences and **(B)** subfamily A/variant 2 sequences. Peptides indicated in **(A)** are putative bactericidal epitopes. In **(B)** positively selected sites are indicated. Note: subfamily A/variant 2 differs in length from subfamily B/variant 1 by +4 bp (e.g., Glu151 is equivalent to Glu147 in variant 1). Reproduced from Brehony et al. (14) without modification under CC BY.

Given the known diversity of meningococcal protein antigens, assessment of the levels of diversity of these new vaccine components, especially the leading candidate fHbp (LP2086) formed a major part of the preclinical studies. Based on the analysis of deduced peptide sequences, the Pearl River group (studying “LP086”) proposed two subfamilies of the protein, A and B, whereas the Siena group (studying “GNA1870” later called fHbp), using a similar analysis of a different meningococcal collection, proposed three subvariants (1, 2, and 3, with subvariants 2 and 3 more closely related to each other) (85). As further sequencing was performed of this antigen, it became necessary to cross-reference and unify the sequence nomenclatures between the two typing schemes and a single nomenclature was proposed (14) (Figure 5), along with a web accessible database enabling the cross-referencing of the various nomenclature schemes (https://pubmlst.org/neisseria/fHbp/). At the time of writing (September 2018), a total of 1,157 peptide sequence variants of this protein were described on the database.

As with the capsular polysaccharide-conjugate vaccines, it was impractical to conduct phase III efficacy studies on these novel “serogroup B substitute” vaccines. Further, the correlates of protection were less well-established than those for the capsular polysaccharide vaccines, where bactericidal assays using blood samples from vaccinees were employed (3). This was a particular problem in assessing the breadth of coverage of these vaccines, given the diversity of meningococcal protein antigens. This prompted the development of indirect assessments of coverage: the Meningococcal Antigen Typing System (MATS) assay for Bexsero®, which incorporates sequence data from the *porA* gene (86); and the Meningococcal Antigen Surface Expression (MEASURE) Assay for Trumenba^®^ (87). Both vaccines were licensed and used based on phase II clinical studies and studies using these assays (88, 89).

**Figure 6:**
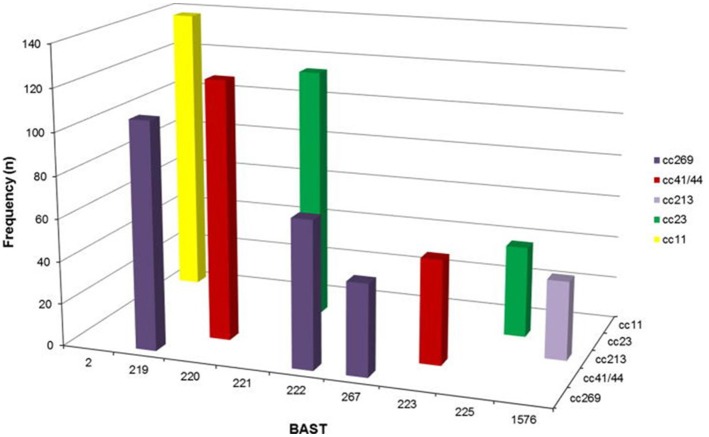
Frequency distribution of Bexsero Antigen Sequence Typing (BAST) by clonal complex for the nine most frequently occurring BASTs (*n* = 775/2,016, 39.4%) in invasive meningococcal disease isolates in the epidemiological years 2010/2011 to 2013/2014. BASTs shown on the x axis are structured by clonal complex (z axis) for a proportion of frequently occurring dominant clones circulating in Great Britain and Ireland from 2010/2014, for example BAST-2 is only found in isolates of clonal complex 11. Clonal complex 269 has three predominant BASTs, 219, 222, and 267 not found in other clonal complexes. Reproduced without modification from Brehony et al. (91) under CC BY.

Pre- and post-introduction assessment of the coverage of these vaccines is an important component of assessing their likely and continued efficacy, especially for an organism as diverse and dynamic as the meningococcus. An efficient way to achieve this is by the extraction of antigen gene sequences from genome data collected as part of routine surveillance. The PubMLST website, which hosts data for the MRF-MGL and a number of other important global isolate collections, indexes all known genes and these can be flexibly grouped into “schemes,” which are groups of genetic loci that are analyzed together for typing or functional purposes (13, 90). It was straightforward to combine the typing schemes for the various antigens in Bexsero® into a Bexsero® Vaccine Antigen Typing Scheme (BAST) (91), which enabled the assessment of the changing prevalence of the vaccine antigen variants in the UK and Ireland (91, 92) (Figure 6). Approaches such as this have great potential for supporting vaccine development implementation and monitoring into the future.

## Future Prospects

As demonstrated by the examples outlined above, nucleotide sequence data, ranging from single gene fragments from individual bacterial specimens to whole completed genomes that are representative of populations, have many applications in vaccinology. These data are particularly useful in the rapid and cost-effective characterization of bacterial isolates. Combining such data with population and evolutionary analyses generates many informative inferences; however, whilst this complements data on the interaction of bacterial components with the host immune system, sequence analyses cannot wholly replace immunological studies. In an era where nucleotide sequences are low-cost commodities, the important advances of the future will depend on the interpretation and open-access dissemination of these data. In addition to novel statistical genetic techniques and integration with phenotypic data, the implementation of visualization tools is likely to be important in the further exploitation of this rich source of biological information.

## Author Contributions

The author confirms being the sole contributor of this work and has approved it for publication.

### Conflict of Interest Statement

As an employee of the University of Oxford, MM has, over the past 20 years, undertaken contract research and consultancy for, and has been paid expenses and honoraria by, companies involved in vaccine development including GSK, Chiron, Novartis, Wyeth, and Pfizer.
